# Three algorithms and SAS macros for estimating power and sample size for logistic models with one or more independent variables of interest in the presence of covariates

**DOI:** 10.1186/1751-0473-9-24

**Published:** 2014-11-15

**Authors:** David Keith Williams, Zoran Bursac

**Affiliations:** Department of Biostatistics, University of Arkansas for Medical Sciences, Little Rock, AR USA; Division of Biostatistics and Center for Population Studies, Department of Preventive Medicine, College of Medicine, University of Tennessee Health Science Center, Memphis, TN USA

**Keywords:** Logistic regression, Power, Sample size

## Abstract

**Background:**

Commonly when designing studies, researchers propose to measure several independent variables in a regression model, a subset of which are identified as the main variables of interest while the rest are retained in a model as covariates or confounders. Power for linear regression in this setting can be calculated using SAS PROC POWER. There exists a void in estimating power for the logistic regression models in the same setting.

**Methods:**

Currently, an approach that calculates power for only one variable of interest in the presence of other covariates for logistic regression is in common use and works well for this special case. In this paper we propose three related algorithms along with corresponding SAS macros that extend power estimation for one or more primary variables of interest in the presence of some confounders.

**Results:**

The three proposed empirical algorithms employ likelihood ratio test to provide a user with either a power estimate for a given sample size, a quick sample size estimate for a given power, and an approximate power curve for a range of sample sizes. A user can specify odds ratios for a combination of binary, uniform and standard normal independent variables of interest, and or remaining covariates/confounders in the model, along with a correlation between variables.

**Conclusions:**

These user friendly algorithms and macro tools are a promising solution that can fill the void for estimation of power for logistic regression when multiple independent variables are of interest, in the presence of additional covariates in the model.

**Electronic supplementary material:**

The online version of this article (doi:10.1186/1751-0473-9-24) contains supplementary material, which is available to authorized users.

## Introduction

The purpose of this work is to propose and demonstrate the *%LRpowerCorr10* algorithm (and two related algorithms) which estimates power and sample size for logistic models in settings where one or more predictors are of primary interest (Additional file 1). Additionally, covariates (confounders) may be present in the model. All the potential predictors can have a specified correlation structure and may be from a mixture of different univariate distributions, namely normal, uniform, and binomial. The user inputs several conjectured attributes including sample size, correlation, and odds ratios for association between independent variables and the outcome, and the result is an estimate of power. Two other related algorithms are also described. In short, a second algorithm, *%Quickpower* provides the inverse of *%LRpowerCorr10*, that is, sample size for a given power. A third algorithm, *%LRpowerCorr10C* provides an approximate power curve for a given range of sample sizes.

## Background

The motivation for this work stems from methods that are in use to estimate power and sample size for standard linear regression models [1–4]. The MULTREG statement within SAS PROC POWER [1, 5] allows the investigator to determine the power to detect significance for a model with set of primary predictors of interest in the presence of covariates which are included in the model, but not of primary interest. For example, suppose an investigator proposes a linear model with four total predictors X1, X2, X3, and X4 but is primarily interested in X1 and X2 while controlling for X3 and X4. To power this setting the full model would be:


while the reduced model would be:


This corresponds to testing the null hypothesis:


in the full model. In the best case scenario to accurately estimate power, we would like to know the difference in the R-square of the full model and R-square for the reduced model. As an illustration, the short SAS code below would return a power value of 0.864.

### SAS code



The MULTREG statement works nicely, but requires estimates of R-squared that investigators may not know in advance. However, with some matrix algebra investigators can arrive at estimates for the R-squared for both the full and reduced models if they can provide a set of assumed correlations between each predictor X and Y, along with assumed correlations among each of the variables X. The details follow. R-squared can be expressed with the matrix expression,


where *ρ*_*yx*_ is the 1 x p vector of simple correlations between each of the individual p predictors and the response variable y, and  is the inverse of the p x p correlation matrix among each of the predictors. Next, one can calculate R-square for the reduced model by doing the identical calculation with the removal of the predictors of interest from the rows of *ρ*_*yx*_ and the rows and columns of . An example of these calculations is as follows,


where the leading row vector is the set of simple correlations of Y with each of the four predictors X1, X2, X3, and X4. The middle matrix is the correlation of all four predictors X1, X2, X3, X4 among themselves, and the last column vector is the transpose of the leading vector. The correlation values are from a particular data set and are intended for demonstration. If we are interested in investigating power for X1 and X2 while controlling for X3 and X4 we would use the calculation,


in which it can be seen that the first two columns of the leading vector and the first two rows and columns of the middle matrix (which correspond to X1 and X2) have been omitted. The difference in these two calculations results in,


which represents another approach to providing the difference in R-squares, a quantity needed in order to calculate power for this regression model setting. A corresponding set of calculations can be done for any size set of p predictors with a set of predictors of interest with the compliment of this set representing the predictors that are serving for controls. It is a reasonable approach in that researchers in many instances will have some idea of the simple correlations among the response and the predictors before their study, so this approach does have its merit.

Our objective was to provide a power estimation method for logistic regression settings that work in a somewhat corresponding manner to the matrix approach above for ordinary least squares regression. Currently, all the software the authors are aware of (e.g., SAS, PASS, nQuery), estimate logistic model power of only one predictor of interest in the presence of some number of other covariates [1, 4]. A well written and documented SAS macro intended for this scenario is the *%PowerLog* macro [6]. The *%PowerLog* macro works nicely for this scenario but is not able to estimate power for a corresponding setting as was discussed above, that is, having more than one predictor of interest in a model controlling for other covariates. Furthermore, all these methods and software require inputs that are not always user friendly to researchers and require some initial knowledge of relationships as well as preliminary calculations. The proposed approach has the user providing the conjectured odds ratios associated with each predictor and the binary outcome, in addition to the correlations among all the predictors which seems more intuitive to many users. This approach has merit since the values of regression coefficients are equal to the natural log of the odds ratio. Demidenko et al. [7], published a similar approach using odds ratios, and currently provides an online applet (http://www.dartmouth.edu/~eugened/power-samplesize.php), however, it is applicable only to one independent variable in the presence of one confounder. Therefore our proposed methods and SAS tools extend the currently available methodology so that one can power studies for multiple independent variables of interest, in the presence of multiple covariates or confounders. In the next section we outline our algorithm to estimate power for a given sample size in this manner. It is worth noting that the SAS macros LRPowerCorr10, LRPowerCorr10C, and QuickPower that use the algorithm can accommodate up to 10 predictors, X1-X10. Another feature of the SAS macros is that X1 and X2 are binomial predictors, X3-X6 are uniform (-3,3) predictors, and X7-X10 are standard normal (0,1) predictors. The investigator may use any or all of these that may fit their setting.

## Methods

### *LRpowerCorr10*algorithm steps

1. Define OR1-OR10 (the odds ratio associated with predictors X1-X10), AVEP (the average proportion of outcome Y = 1 when covariates X1 – X10 equal zero), and **P**, the correlation matrix of the predictors.
2. Create **W**, a n x 10 data matrix by simulating n rows of ten univariate distributions with given means and standard deviations.
3. Create **Z** by standardizing each element of **W** by subtracting the appropriate column mean and dividing by the corresponding standard deviation.
4. Define **P** the correlation matrix of the 10 predictor variables. Calculate the Cholesky decomposition of **P**, that is, the matrix **U** such that U’U = **P**.
5. Calculate **X** = **Z U’**
6. Multiply each element of **X** by its column’s standard deviation and then add the column appropriate mean.
7. Calculate: logit = ln(AVEP/(1-AVEP)) + ln(OR1)X1 + … + ln(OR10)X10. Next calculate phat = exp(logit)/(1 + exp(logit)). Phat represents the probability that Y = 1 for a particular case.
8. If phat is less than or equal to a random uniform (0,1) draw then Y = 1, otherwise Y = 0. This step is needed to convert a phat probability to an appropriate binary value in order to run PROC LOGISTIC.
9. Using SAS PROC LOGISTIC, fit the full model y = X1 X2 X3 X4 X5 X6 X7 X8 X9 X10 and save the -2 log likelihood value.
10. Using SAS PROC LOGISTIC, fit the reduced model which has the predictors of interest omitted from the full model and save the -2 log likelihood value.
11. Save the difference in the full and reduced model -2 log likelihood values (likelihood ratio test; LR) [8] and determine if this value is greater or equal to the appropriate critical value. If this is the case, record this single simulation run as a ‘rejection’.
12. Repeat steps 1–10 *m* times and tabulate the proportion of rejections. This proportion will be the estimate of the power for the specified scenario. Experience suggests that m =100 is adequate to quickly evaluate scenarios. When a precise final power estimate is required, m =1000 provides an estimate with a standard error of about 0.01.

### The *%LRpowerCorr10*SAS macro

The user must define several variables as shown in Table 1. The macro variable SAMPLESIZE corresponds to the sample size that the macro is evaluating. NSIMS is the number of simulation runs required by the user, while P is the correlation among all of the predictors. AVEP is the average proportion of ‘yes’ responses (Y = 1) when all the predictor values are theoretically equal to zero. OR1 through OR10 are odds ratio values associated with the predictor variables X1-X10. X1 and X2 are binomial variables with probability of success defined by PCX1 and PCX2. X3 through X6 are uniformly (-3,3) distributed and X7 through X10 follow the standard normal distributions. The FULLMODEL macro variable has the user list the predictor variables in the full model. It should be noted that this is the literal script that is placed to the right of the equal sign in the model statement of the PROC LOGISTIC routine inside the macro, so care should be taken for accuracy. In a like manner, the REDUCEDMODEL variable is the list of predictors left in the model after the terms of interest are removed from the FULLMODEL list. ALPHA is the level of significance and DFTEST is the degrees of freedom for the likelihood ratio test [8]. This will correspond to the number of predictor terms of interest, that is, the difference in the number of terms in the FULLMODEL and REDUCEDMODEL lists. Users should provide a value for OR1 through OR10. If particular predictor variables are not used in a power calculation, their corresponding OR should be set to ‘1’ to avoid matrix algebra calculation problems. This point can be seen in practice in the provided examples.

**Table 1:** **LRpowerCorr10 macro variables**

SAMPLESIZE	The sample size to be evaluated
NSIMS	The number of simulation runs
P	The correlation among the predictors
AVEP	The average number of “1” responses in the samples
OR1	The odds ratio associated with X1 (Binomial)
OR2	The odds ratio associated with X2 (Binomial)
OR3	The odds ratio associated with X3 ( Uni(-3,3) )
OR4	The odds ratio associated with X4 ( Uni(-3,3) )
OR5	The odds ratio associated with X5 ( Uni(-3,3) )
OR6	The odds ratio associated with X6 ( Uni(-3,3) )
OR7	The odds ratio associated with X7 ( N (0,1) )
OR8	The odds ratio associated with X8 ( N (0,1) )
OR9	The odds ratio associated with X9 ( N (0,1) )
OR10	The odds ratio associated with X10 ( N (0,1) )
FULLMODEL	The predictor terms in the full model among X1-X10
REDUCEDMODEL	The predictor terms in the reduced model among X1-X10
ALPHA	The significance level of the testing
DFTEST	The degrees freedom of the testing
PCX1	The probability of success for X1
PCX2	The probability of success for X2

### The *%QuickPower SAS*macro

The *%QuickPower* macro outputs a sample size needed to achieve user specified power. The user inputs the exact same set of input variables as *%LRpowerCorr10* except SAMPLESIZE. Instead of SAMPLESIZE user inputs desired POWER, for instance 0.8. In addition user inputs number of terms in the full model (NTERMSFULL) right after the reduced model is specified. This macro allows the user to get a quick approximate idea of what sample size will be required for a given scenario. It is sometimes beneficial to run this macro first to get a ball park idea of required sample size, followed by *%LRpowerCorr10* macro, instead of repeating simulations in order to reach desired power.

### The *%LRpowerCorr10C*SAS macro

The *%LRpowerCorr10C* macro creates an approximate power curve for a user supplied interval of sample sizes, which can be useful for grants and papers. Instead of SAMPLESIZE or POWER, user supplies the input variable LOWER and UPPER (desired sample size range) in addition to the rest of the input set for the *%LRpowerCorr10* macro. LOWER is the minimum value on the horizontal axis of the output graph and UPPER is the maximum value.

### User notes and cautions

1. To minimize numerical problems that can arise from complete separation, ensure that the product n*AVEP (the product of the sample size and the average proportion where Y = 1), as well as the product n*(1 – AVEP), is at least 10.
2. Caution and thought should go into the value(s) of OR and average sample proportion being evaluated for multiple logistic regression model power. If one evaluates OR3 = 2 along with AVEP = 0.1 in the setting in which the X3 is from the uniform (-3, 3) distribution, roughly implies that the P(Y = 1) approximately doubles for each one unit increase in X3, which is not always reasonable. Thoughtful values of conjectured odds ratios are vital to the macro’s usefulness to supply meaningful sample size and power values.
3. The *%LRpowerCorr10* macro uses the LR chi-square test statistic to evaluate power [8]. Some other power approaches use the Wald chi-square test for the power evaluation [7]. These statistics have asymptotically the same type I error and are locally equivalent, however globally they are different tests so while close, they don’t always produce exactly same estimates of sample size and/or power [7]. Most statisticians would agree that the LR chi-square is generally a bit more sensitive and this implies that if one compared equivalent scenarios, it is likely that the LR chi-square approach would be slightly more powerful, but still very close.

## Application and results

In the first example we demonstrate *%LRpowerCorr10* macro by specifying a sample of 700 for a scenario with four independent covariates of interest X1-X3 and X7, two of which are binary (X1 and X2), one uniform (X3) and one standard normal (X7), with hypothesized ORs of 1.5, 1.5, 1.1 and 1.1 in bold font below, respectively. Full model also includes 4 additional covariates, X4, X8-X10. Hypothesized correlation between variables is 0.2, and P(Y = 1) = 0.1.

### *%LRPowerCorr10*macro example commands



After running the algorithm described above 1000 times, macro yields the power estimate of 80% with 95% CIs ranging from 77% to 82%.

### *%LRPowerCorr10*macro example output



In the second example we show how to use *%Quickpower* macro. The purpose of this macro is to provide a user with a quick sample size estimate for a given scenario. Below we specify the same model as in the first example with the same ORs. Instead of a sample size in this case we input the desired power, which is 0.8 in bold font. This macro also requires the number of variables in the full model which is 8, also in bold font. Other parameters remain the same.

### *%QuickPower*macro example commands



The *%Quickpower* macro estimate of the sample size was 671. Within *%Quickpower* macro call this estimate was inserted into *%LRpowerCorr10* macro and after running the algorithm 1000 times, macro yields the power estimate of 77% with 95% CI ranging from 74% to 80%. Since the sample size of 671 appears to be slightly underpowered we could adjust it to 700 or higher as needed, and rerun the *%LRpowerCorr10* to get the power in desired range.

### *%QuickPower*macro example output



In the third and final example we present the use and the results of the *%LRpowerCorr10C* macro which provides an approximate power curve for the user specified range of sample sizes. Again we use the same scenario as above for consistency purposes, and we input the sample size range from 600 to 1100, in bold font below. The rest of the parameters remain the same.

### *%LRPowerCorr10C*macro example commands

**Figure 1:**
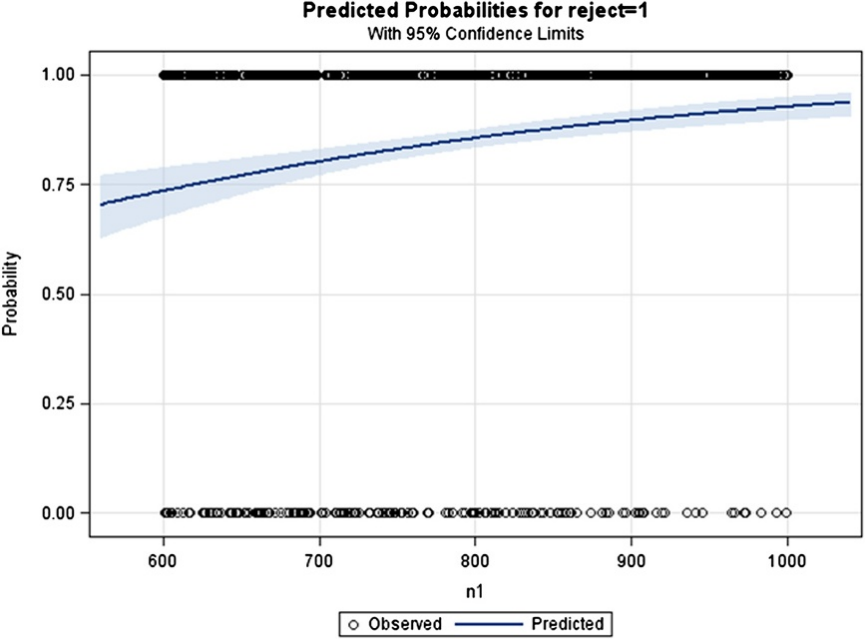
***%LRpowerCorr10C*** **macro example output.**

The resulting figure below shows that a sample of 600 has a power of slightly below 75%, and as sample approaches 1100 power reaches 95%. Sample size of 700 has approximate power of 80%, therefore based on ones needs, desired sample size can be gauged (Figure 1).

## Conclusions

The *%LRpowerCorr10* macro and the algorithm it is based on (as well as other two algorithms proposed in this paper), shows promise to fill a void for estimating power for multivariable logistic models when multiple covariates are of interest. It is able to match the approach that researchers use for multiple regression when estimating the power of a model in which one or more predictors are of interest while controlling for a number of other variables or confounders. There doesn’t exist another tool on the market quite like this one, which allows us to power multiple independent covariates in the presence of additional variables in the model. Furthermore, unlike some other tools, inputs for the proposed algorithms are more intuitive in the form of odds ratios that most researchers are familiar with, and can test several possible magnitudes based on their assumptions. It allows us to specify the amount of correlation among all the predictors and attempt to match real data analysis settings that researchers commonly encounter.

## Electronic supplementary material

Additional file 1: **Text file that contains the three SAS macros discussed in this manuscript.** (TXT 17 KB)
